# Neurotrophins Regulate Bone Marrow Stromal Cell IL-6 Expression through the MAPK Pathway

**DOI:** 10.1371/journal.pone.0009690

**Published:** 2010-03-15

**Authors:** Fariba Rezaee, Stephanie L. Rellick, Giovanni Piedimonte, Stephen M. Akers, Heather A. O'Leary, Karen Martin, Michael D. Craig, Laura F. Gibson

**Affiliations:** 1 Department of Pediatrics, West Virginia University School of Medicine, Morgantown, West Virginia, United States of America; 2 Department of Neurobiology and Anatomy, West Virginia University School of Medicine, Morgantown, West Virginia, United States of America; 3 Mary Babb Randolph Cancer Center, West Virginia University School of Medicine, Morgantown, West Virginia, United States of America; 4 Department of Microbiology and Immunology, West Virginia University School of Medicine, Morgantown, West Virginia, United States of America; Katholieke Universiteit Leuven, Belgium

## Abstract

**Background:**

The host's response to infection is characterized by altered levels of neurotrophins and an influx of inflammatory cells to sites of injured tissue. Progenitor cells that give rise to the differentiated cellular mediators of inflammation are derived from bone marrow progenitor cells where their development is regulated, in part, by cues from bone marrow stromal cells (BMSC). As such, alteration of BMSC function in response to elevated systemic mediators has the potential to alter their function in biologically relevant ways, including downstream alteration of cytokine production that influences hematopoietic development.

**Methodology/Principal Findings:**

In the current study we investigated BMSC neurotrophin receptor expression by flow cytometric analysis to determine differences in expression as well as potential to respond to NGF or BDNF. Intracellular signaling subsequent to neurotrophin stimulation of BMSC was analyzed by western blot, microarray analysis, confocal microscopy and real-time PCR. Analysis of BMSC Interleukin-6 (IL-6) expression was completed using ELISA and real-time PCR.

**Conclusion:**

BMSC established from different individuals had distinct expression profiles of the neurotrophin receptors, TrkA, TrkB, TrkC, and p75^NTR^. These receptors were functional, demonstrated by an increase in Akt-phosphorylation following BMSC exposure to recombinant NGF or BDNF. Neurotrophin stimulation of BMSC resulted in increased IL-6 gene and protein expression which required activation of ERK and p38 MAPK signaling, but was not mediated by the NFκB pathway. BMSC response to neurotrophins, including the up-regulation of IL-6, may alter their support of hematopoiesis and regulate the availability of inflammatory cells for migration to sites of injury or infection. As such, these studies are relevant to the growing appreciation of the interplay between neurotropic mediators and the regulation of hematopoiesis.

## Introduction

Neurotrophins are a family of proteins which are best characterized by their modulation of survival, differentiation and apoptosis of cells in the nervous system. This family includes NGF, BDNF, neurotrophin 3 (NT-3), and neurotrophins 4/5 (NT-4/5)[1]. Neurotrophins signal through the high-affinity tropomyosin receptor kinase (Trk) receptors, TrkA, TrkB, TrkC, and the low-affinity receptor, p75^NTR^, a member of the tumor necrosis factor receptor family[1], [2].

NGF is a survival factor essential for a large number of neuronal and non-neuronal cell types. The importance of neurotrophin signaling is highlighted by neurodegenerative conditions such as Alzheimer's disease, in which there is a dysregulation of pathways modulated by neurotrophic factors[3], [4]. In addition to its role in neurological pathways, neurotrophin signaling has an impact on innate and adaptive immunity[5]. Alteration of NGF has been documented in autoimmune inflammatory diseases including multiple sclerosis[6], psoriasis[7], systemic lupus erythematosus[8] and rheumatoid arthritis[9]. Traumatic brain injury[10], neuroectodermal tumors[11] and endocrine disorders[12] are a few examples of many conditions also associated with increased neurotrophins. A positive correlation between NGF level and allergic asthma, airway hyperactivity, total IgE and the number of eosinophils in the serum has also been noted[13]. These observations suggest that neurotrophins may mediate hematopoietic responses to several clinically relevant conditions. Importantly, NGF has the potential to act systemically on distant organs, including the bone marrow which serves as the primary site of postnatal hematopoiesis[14], [15].

BMSC provide the structural and physiological support for hematopoietic cell survival, proliferation and differentiation. Resident stem and immature hematopoietic progenitor cells mature under the influence of the bone marrow microenvironment to functional, mature cells of diverse lineages[14], [15]. As such, exposure of this microenvironment to circulating neurotrophins, cytokines and growth factors has the potential to alter its function, resulting in the generation of hematopoietic populations that are markedly different than those in healthy individuals.

In the current study, a cytokine that was consistently and significantly increased in BMSC exposed to NGF or BDNF was Interleukin-6 (IL-6). IL-6 is a multifunctional cytokine[16] modulated by other factors including IL-1, TNF-α, growth factors, hormones, and viral or microbial products[17]–[19]. Dysregulation of IL-6 production has been reported in the pathogenesis of several autoimmune diseases including rheumatoid arthritis, systemic-onset juvenile chronic arthritis, autoimmune encephalomyelitis, psoriasis, antigen-induced arthritis, and Systemic Lupus Erythematosus[16], [20]–[22]. IL-6 is a critical factor for hematopoiesis through regulation of the entry of hematopoietic stem cells into the cell cycle, proliferation of cells committed to the myeloid and lymphoid lineage, and maturation of B-cells into antibody producing cells[16], [23]–[26]. Increased IL-6 expression in transgenic mice results in massive polyclonal plasmacytosis and malignant plasmacytoma[26]. In contrast, a reduction in hematopoietic progenitor cell support has been reported by IL-6 deficient bone marrow stromal cells[27]. These observations suggest that changes in IL-6 levels could impact on the development of hematopoietic populations available to participate in inflammatory responses with the novelty of our current study derived from consideration of the potential of systemic neurotrophic factors to modulate IL-6 in the marrow microenvironment through direct stimulation of BMSC.

Depending upon the cellular context, IL-6 transcription has been documented to be influenced by both NF-κB and MAPK (mitogen-activated protein kinase) cascades subsequent to NGF stimulation[28], [29]. Studies have shown that NGF activates NF-κB in rat pheochromocytoma PC12 cells[30]. NF-κB is sequestered in the cytoplasm by the IκB family of proteins which become phosphorylated, and degraded by the proteasome with subsequent NF-κB translocation to the nucleus[31]. As a transcription factor involved in the control of inflammatory responses, cellular growth, and apoptosis[31], NF-κB is involved in the pathology of several diseases, including cancers, arthritis, chronic inflammatory bowel disesse, asthma and neurodegenerative diseases[32]–[36]. Neurotrophin stimulation of the MAPK pathway has been documented in the PC12 cell line[37], dorsal root ganglia, and transient receptor potential vanilloid 1 (TRPV1) (the capsaicin receptor) model[38]. Activation of the TrkA receptor by NGF in airway smooth muscle cells also leads to activation of the MAPK cascade including p38 MAPK, and extracellular-regulated protein kinase 1/2 (ERK1/2)[39]. Following stimulation, the MAPK family of proteins activate several downstream factors involved in regulating inflammation[37]–[39].

Based on the diverse set of pathologic conditions associated with dysregulated neurotrophic factors, many of which involve inflammation as a central feature, we investigated the effects of NGF and BDNF on BMSC function as a critical influence on regulation of hematopoietic cell development.

## Results

### BMSC have distinct expression profiles for TrkA, TrkB, TrkC and p75^NTR^

**Figure 1:**
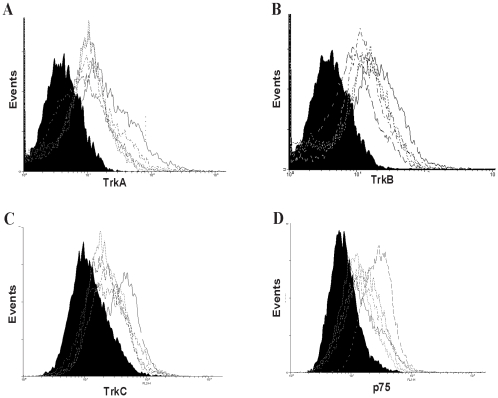
BMSC express neurotrophin receptors. BMSC established from different individuals were stained for high affinity receptors (A) TrkA, (B) TrKB, (C) TrkC and the low affinity receptor (D) p75NTR with specific antibodies followed by flow cytometric evaluation. BMSC expressed distinct profiles of neurotrophin receptors. Isotype matched controls are indicated by the solid histogram.

To determine the expression pattern of neurotrophin receptors by BMSC, flow cytometry analysis of BMSC with antibodies specific for, TrkA, TrkB, TrkC and p75^NTR^ was performed. While the levels of expression for a given receptor varied between stromal lines derived from different individuals, all BMSC lines used in this study expressed both the high and low affinity receptors to respond to neurotrophins (Figure 1).

### BMSC demonstrate Akt phosphorylation following NGF or BDNF exposure

**Figure 2:**
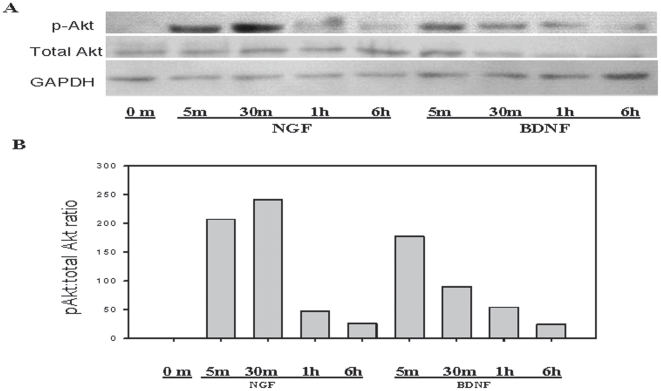
BMSC Akt phosphorylation increases following NGF or BDNF exposure. Following treatment with NGF or BDNF, BMSC were lysed and protein was resolved by SDS-PAGE and transferred to nitrocellulose membranes. (A) Membranes were probed with antibodies specific for phospho-Akt and total Akt; GAPDH was used as a lane loading control. (B) Densitometry demonstrates an increase in phospho-Akt:Total Akt following NGF or BDNF exposure.

Activation of Akt occurs in response to a variety of stimuli, and relevant to the current study, is a well documented consequence of neurotrophin stimulation[40]. Therefore, while not a investigative focus in this work, the phosphorylation of Akt was utilized simply as a read-out to determine if BMSC could respond to neurotrophin binding to the Trk receptors. Phosphorylation of Akt occurred rapidly following BMSC exposure to 100 ng/ml of NGF or BDNF. NGF treated BMSC Akt phosphorylation was detected as early as 5 minutes with the peak signal occurring at 30 minutes. Phosphorylated Akt began to diminish by 1 hour, and minimal signal was detected after 6 hours of exposure to NGF. In BDNF treated cells, peak phosphorylation of Akt occurred at 5 minutes and gradually diminished over 6 hours (Figure 2).

### Exposure to NGF or BDNF increases IL-6 gene expression in the BMSC

**Figure 3:**
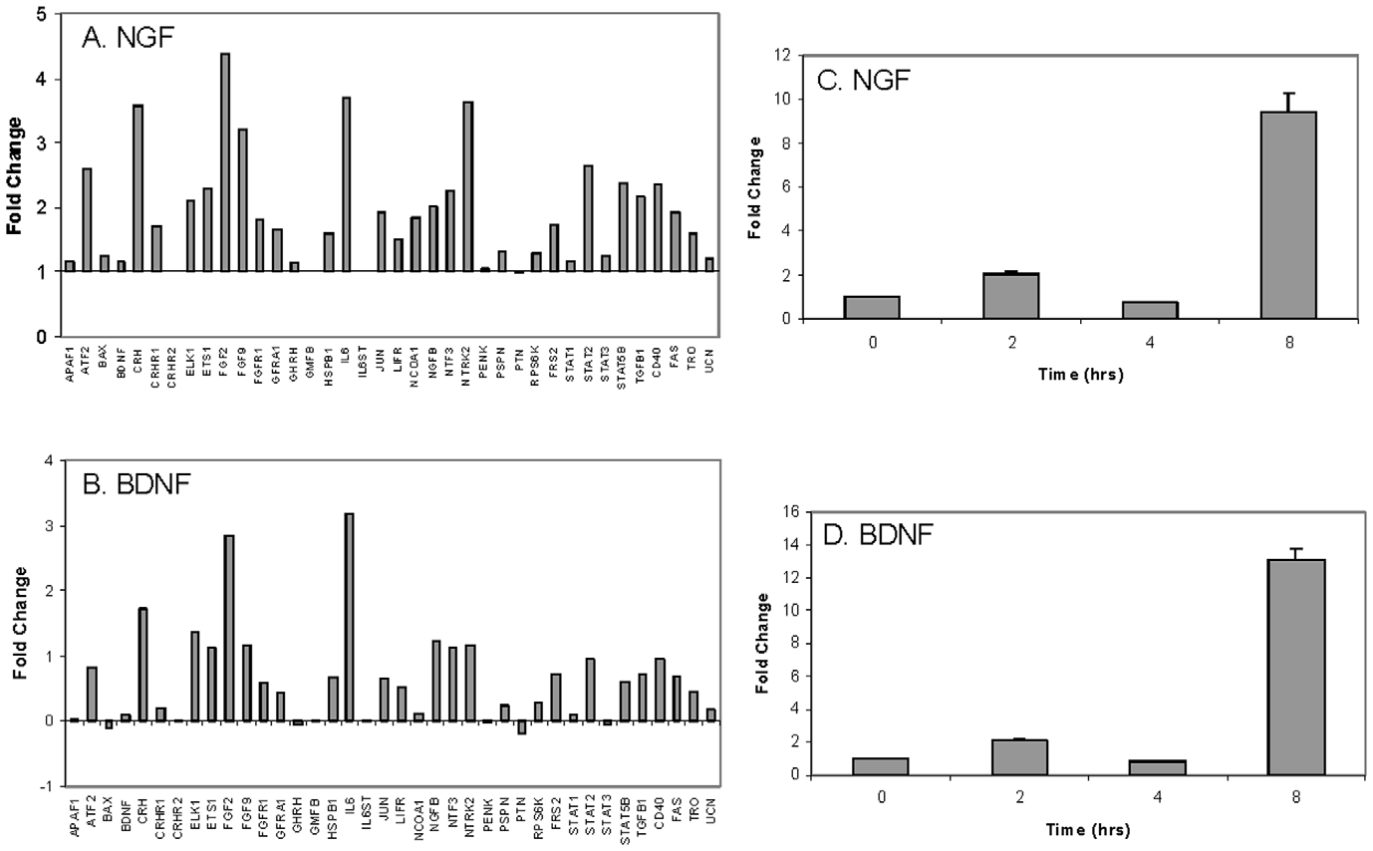
NGF or BDNF changes the gene expression profile of BMSC and increases IL-6 mRNA. BMSC were treated with 100 ng/ml of (A) NGF or (B) BDNF for 18 hours. The cells were collected, RNA was isolated, and microarray analyses were performed. Graphs summarize the panel of gene expression changes in the BMSC treated with NGF or BDNF compared to untreated control cells. BMSC were treated with 100 ng/ml of (C) NGF or (D) BDNF for 2, 4 and 8 hours. The cells were collected, RNA was isolated and real time PCR was performed with the one-step QuantiTech SYBR Green kit as instructed by the manufacturer. BMSC IL-6 expression in response to NGF or BDNF exposure compared to untreated control cells is shown. Expression was normalized to the housekeeping gene *GUSB*. Statistical analysis was completed by ANOVA (*P*≤0.0001) with significance indicated by an asterisk.

To investigate the cytokine pattern of BMSC exposed to neurotrophic factors, RNA isolated from BMSC exposed to NGF and BDNF was analyzed by microarray as described. While many genes were increased, and fewer decreased, following NGF or BDNF exposure, with a cut-off of 3 fold-change compared to untreated controls, Fibroblast Growth Factor-2 (FGF2) and IL-6 emerged as those genes markedly upregulated following NGF or BDNF treatment in this panel (Figure 3A–B). In addition, the BDNF receptor (NTRK2) and CRH1 gene expression increased following NGF exposure, but did not meet the threshold cutoff in cells exposed to BDNF. IL-6 was chosen for further investigation to determine the signaling pathway that may underlie altered expression based on its well characterized role in hematopoiesis.

To validate our IL-6 microarray data, relative transcript levels were determined by quantitative real-time PCR. Consistent with the microarray data, IL-6 mRNA increased in BMSC after exposure to both NGF and BDNF (Figure 3C-D).

### BMSC exposed to NGF or BDNF demonstrate increases in IL-6 protein consistent with gene expression changes

**Figure 4:**
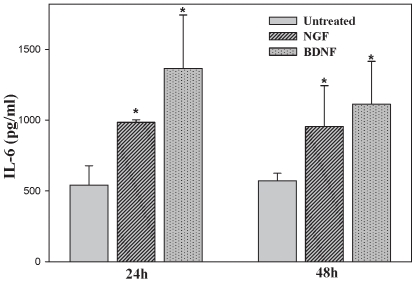
BMSC treatment with NGF or BDNF increases IL-6 protein. BMSC were exposed to 100 ng/ml of NGF or BDNF for 24 and 48 hours. The supernatant was then collected and an IL-6 ELISA was performed. An increase in BMSC IL-6 protein was noted in all treatment groups. Statistical analysis was completed by ANOVA (P≤0.01) with significance indicated by an asterisk.

To evaluate effects of NGF or BDNF on IL-6 protein, BMSC were serum deprived (1% FBS) for 24 hours and subsequently exposed to 100 ng/ml of NGF or BDNF for 24 or 48 hours in triplicate. An IL-6 ELISA was performed on the supernatant collected from each time point to determine changes in the production of IL-6 protein. Consistent with the up-regulation of IL-6 message, exposure of BMSC to NGF and BDNF increased the level of IL-6 protein (Figure 4). To determine the optimal concentration of neurotrophins needed to induce NGF or BDNF signaling, resulting in IL-6 protein increase, a dose response curve was completed, using an IL-6 ELISA with doses of neurotrophins ranging from 0–100 ng/ml. A MTT assay was used to determine the concentrations of neurotrophins used were not toxic to the BMSCs (data not shown). To determine if there is a role for the sIL-6R in our model, BMSC were left untreated or stimulated with NGF or BDNF (10–100 ng/ml) for 24 hours and a sIL-6R ELISA was completed. There was no detectable sIL-6R present in the untreated cells or in those cells treated with NGF or BDNF (data not shown).

### Neurotrophins do not induce NF-κB signaling in BMSC

**Figure 5:**
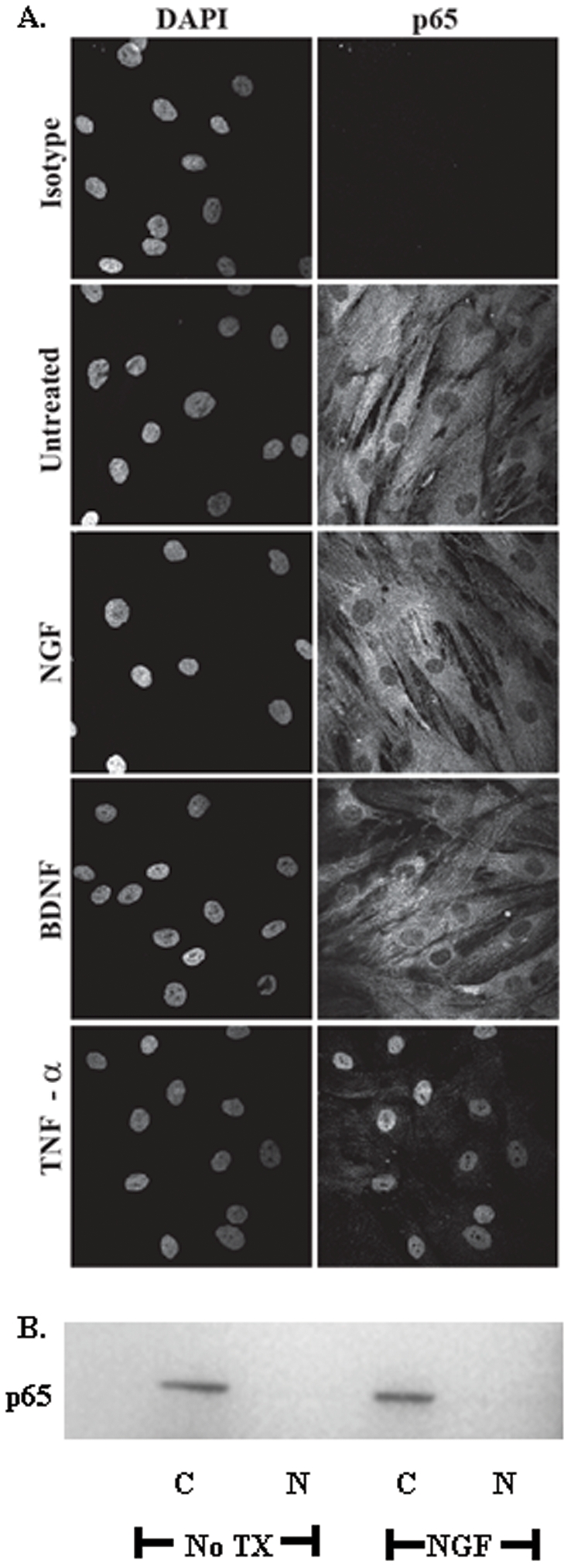
Neurotrophins do not induce NF-κB signaling in BMSC. BMSC were treated with 100 ng/ml of NGF, BDNF, or TNFα for 5, 30 and 60 minutes. Following treatment, BMSC were fixed and probed with antibodies specific for NFκB p65 or its matched isotype control. Analyses of samples by confocal microscopy indicate cytoplasmic p65 in untreated control and translocation of p65 to the nucleus upon stimulation with a known positive stimulus, TNF-α (A). The subcellular localization of p65 did not change in response to NGF or BDNF. Representative images from 30 minutes exposure to stimuli are shown (original magnifications ×40). (B) BMSC were left untreated or treated with NGF (100 ng/ml) for 30 min. and subcellular fractionation and western blot analysis completed with p65 specific antibodies. Treatment of BMSC with NGF did not change the subcellular localization of p65.

In neurons, it has been established that neurotrophin stimulation activates signaling through NF-κB[30]. Additionally, NF-κB is a known transcription factor for IL-6[28]. Using immunofluorescence, we determined whether exposure of BMSC to neurotrophins induced nuclear translocation of the p65 subunit of NF-κB. BMSC (one cell line) were exposed to either NGF or BDNF. Subsequent to NGF or BDNF exposure, no nuclear translocation of the NF-κB p65 subunit was noted. In contrast, rTNF-α, which was used as a positive control, induced rapid translocation of the p65 subunit from the cytoplasm to the nucleus in the BMSC (Figure 5A). To confirm the lack of p65 translocation to the nucleus with NGF stimulation, BMSC were left untreated or treated with NGF (100 ng/ml) for 30 min. Following treatment, cellular fractionation was completed and the fractions analyzed by western blot with p65 specific antibodies. There was no detectable p65 in the nuclear fraction of untreated or NGF treated cells, while p65 was readily detected in the cytoplasmic fraction (Figure 5B). GAPDH was used as a fraction contamination control (data not shown).

### NGF and BDNF induce MAPK ERK1/2 signaling pathway

**Figure 6:**
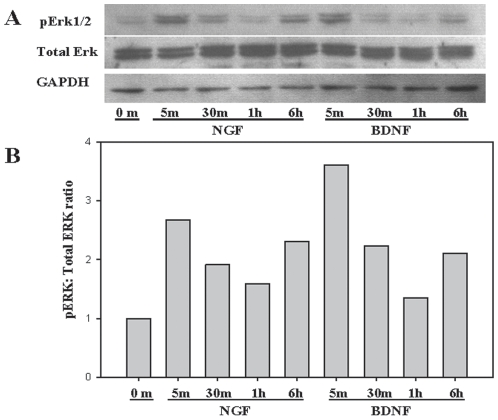
BMSC demonstrate activation of MAPK ERK following NGF or BDNF exposure. Following treatment with100 ng/ml of NGF or BDNF, BMSC were lysed and protein was resolved by SDS-PAGE and transferred to nitrocellulose membranes. (A) Membranes were probed with antibodies specific for phospho ERK1/2 and total ERK; GAPDH was used as a lane loading control. (B) Densitometric quantitation of increase in phosphorylated Erk:Total Erk following NGF or BDNF exposure.

Stimulation of neuronal TrkA with NGF has been shown to activate the MAPK components ERK1/2 and p38 MAPK[39]. To evaluate the role of neurotrophin induced MAPK signaling in BMSC, we analyzed phosphorylation of ERK1/2 as a read out of MAPK activation following BMSC exposure to NGF or BDNF. Phosphorylation of ERK occurred rapidly and transiently in both NGF and BDNF treated groups while no phosphorylation was detected in untreated control cells (Figure 6).

### MAPK ERK1/2 pathway inhibitors blunted IL-6 production following NGF or BDNF exposure

**Figure 7:**
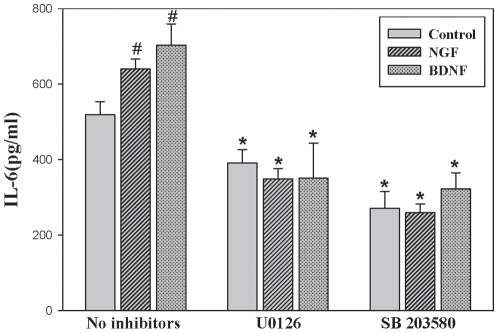
ERK and p38MAPK pathway inhibitors blunted IL-6 protein increase during NGF or BDNF exposure. A MEK1/2 inhibitor, U0126, and p38 MAPK inhibitor SB 203580 was introduced to BMSC cultures 2 hours prior to NGF or BDNF exposure. BMSC were then exposed to 100 ng/ml of either NGF or BDNF for 24 hours and an IL-6 ELISA was performed on collected supernatants. BMSC pre-treated with U0126 or SB 203580 demonstrated greater than 50% decrease in IL-6 protein upon exposure to NGF or BDNF. Statistical analysis was completed by ANOVA (*P*≤0.01) with significance indicated by an asterisk. Groups that included inhibitor were compared to matched group with no inhibitors. BMSC treated with NGF or BDNF without inhibitors had a significant increase in IL-6 protein (p = .001 and p = .002 respectively as determined by a Student's t-test (#)).

As shown previously (Figure 4), treatment with NGF or BDNF increased IL-6 protein in BMSC supernatants. While there was variation in the level of IL-6 protein induction with neurotrophin treatment alone (Figure 4 and Figure 7), the overall increase in IL-6 protein with NGF or BDNF treatment remains consistent. The increase in IL-6 protein with NGF or BDNF stimulation without any inhibitors was statistically significant (p = .001 for NGF and p = .002 for BDNF) (Figure 7). To further investigate the role of ERK1/2 and p38 pathway in neurotrophin induction of IL-6, specific inhibitors targeting both pathways were utilized as discussed in methods. Pre-treatment of BMSC with U0126 and SB 203580 in concentrations held low enough to maintain specificity of inhibition resulted in approximately a 50% decrease of IL-6 upon exposure to NGF or BDNF (Figure 7). To investigate the role of the Akt pathway in neurotrophin induction of IL-6, BMSC were pre-treated as described above with Akt VIII, a specific Akt inhibitor, for 2 hrs prior to stimulation with NGF or BDNF. Akt inhibition did not decrease IL-6 protein detected in BMSC supernatants (data not shown).

## Discussion

The purpose of this study was to determine if BMSC gene expression is responsive to NGF or BDNF as a means by which neurotrophic factors may indirectly influence hematopoiesis. Many *in vivo* and *in vitro* studies have investigated the role of neurotrophins in hematopoiesis. NGF has been demonstrated as an important factor for hematopoietic colony growth and differentiation[41]. Previous work documents its ability to directly influence proliferation, differentiation, and maturation of myeloid progenitors along with induction of migration, survival and activation of mature hematopoietic cells[42]–[44]. NGF is also a chemotactic stimulus for human leukocytes and macrophages[45], [46]. Fewer studies have focused specifically on the response of BMSC to neurotrophic factors as the mechanism by which NGF or BDNF may indirectly influence hematopoiesis. However, there have been previous reports that document the expression of NGF receptors on BMSC[47] as well as the capacity of BMSC to produce and respond to NGF during normal hematopoiesis[48]. The role of BDNF on immune function and hematopoietic cell development is not as well defined as NGF, although impaired B cell development in bone marrow of BDNF deficient mice has been reported[49].

An earlier study demonstrated expression of high affinity neurotrophin receptors by rat marrow stromal cells [50]. In the current study, we have demonstrated expression of both high and low affinity receptors for neurotrophins by human BMSC, with their level of expression varying between BMSC donors (Figure 1A–D). While in some settings signaling through the low affinity receptor p75 following NGF exposure can result in apoptosis[51], in the presence of TrkA the signals are predominantly reported to be anti-apoptotic[52]. Co-expression of both high and low affinity receptors by human BMSC appear to have favored the anti-apoptotic effects of neurotrophin signaling. The neurotrophin receptors are functional as demonstrated by Akt-phosphorylation following stimulation of BMSC by NGF and BDNF (Figure 2A–B). Phosphorylation of Akt, while it may influence survival of BMSC consistent with its role in several settings, was not being investigated in its survival context in our model. Rather, as a well characterized downstream target of neurotrophic signaling it was merely monitored as a measure of functionality of receptors in the absence of a clearly defined signal more immediately stimulated by NGF or BDNF in BMSC.

Through microarray analysis we demonstrated that cytokine gene expression by BMSC changes in response to NGF or BDNF exposure (Figure 3A, B). Any focused panel of gene expression analysis is, by design, not an exhaustive evaluation of all the potential targets that respond to any stimulus. As such, we chose a focused evaluation, and subsequently targeted IL-6 for follow up based on its diverse involvement in innate immunity, hematopoiesis, and inflammatory responses[16]. Consistent with the microarray data, IL-6 gene and protein expression increased after exposure to both NGF and BDNF as determined by real-time PCR and ELISA respectively (Figure 3C, D and Figure 4). In addition to IL-6, FGF2 was increased more than three-fold that of matched controls by both BDNF and NGF. Previous reports have indicated that elevated FGF2 decreased both stromal cell derived factor-1 (SDF-1;CXCL12) mRNA and protein *in vivo* and also diminished the capacity of BMSC to support the expansion of peripheral blood derived stem cells[53]. Uniquely, the Trk B receptor (NTRK2) was increased by NGF, but not BDNF, with the increase reaching our threshold cut-off of three times that of untreated cells. This increase suggests a potential synergistic relationship between these two neurotrophic factors in which an NGF increase may position BMSC cells to more robustly respond to BDNF through modulation of TrkB receptor expression. Consequently, subtle increases in circulating BDNF may have pronounced signaling potential when NGF increases have occurred previously.

The significant role of IL-6 in inflammation has been demonstrated by the diminished ability of IL-6 knockout mice to respond to environmental air pollutants exposure compared to wild-type control[54]. Of specific relevance to the current study, IL-6 overproduction in transgenic animals resulted in an increase in the number of megakaryocytes, plasmacytosis formation and extramedullary hematopoiesis[26], [55], suggesting elevated IL-6 subsequent to circulating NGF or BDNF may contribute to dysregulated immune function. Of note, all reports are not consistent with a clear inflammatory response subsequent to increased NGF, with a recent study suggesting that NGF may, in fact, have anti-inflammatory actions via its regulation of calcitonin gene-related peptide (CGRP) in monocytes[56]. This recent work highlights the necessity of interpreting data within the confines of the specific model being investigated.

Our data do not rule out the possibility of NGF or BDNF acting on gene expression in BMSC through one, or several, intermediate factors. Increased expression or release of Substance-P following neurotrophic stimulation of cells has been described in diverse settings documenting its important role in the hematopoietic-neuro-immune axis in inflammation, as well as normal and malignant hematopoiesis[57]–[62]. Relevant to our model, Substance-P has been shown to participate in upregulation of inflammatory cytokines in fibroblastic cells[63], [64] and more recently in a model of increased IL-6, IL-1β, and TNF-α in a model of burn-induced lung injury[65]. These reports raise the possibility that Substance-P may be an intermediate between neurotrophic factor exposure and altered gene expression in BMSC as well which has not been investigated. Studies that include targeted knock-out of Substance-P will be intriguing, and are required to determine if it is a required mediator of signaling in BMSC. However, regardless of outcome, the biological significance remains that functional changes in a critical component of the marrow microenvironment, BMSC, can be elicited by circulating NGF or BDNF.

Different reports suggest several pathways mediate IL-6 induction and the immunomodulatory effects of neurotrophins based on the stimulus and cell type. Our study demonstrated phosphorylation of ERK1/2 MAPK (Figure 6A, B), with no nuclear translocation of NF-κB detected (Figure 5) following NGF or BDNF treatment of BMSC. Furthermore, we observe that ERK1/2 and p38 MAPK pharmacological inhibitors (U0126 and SB203580) significantly reduced IL-6 production by BMSC during exposure to NGF or BDNF (Figure 7). This observation is consistent with a recently reported model that included LPS stimulated BMSC in which SB-203580 inhibited IL-6 and IL-11 mRNA expression[66]. Our data suggest ERK and p38 MAPK pathways are required for optimal BMSC IL-6 induction by NGF and BDNF, with these pathways likely involved in baseline expression of IL-6 as a reduction in steady state expression was noted with both inhibitors in the absence of any neurotrophic stimulation. The inhibition noted following stimulation was approximately 50% suggesting that other pathways are also involved in NGF and BDNF stimulated IL-6 production. While a dose response of increased inhibitor concentration could be completed to determine if more pronounced inhibition can be achieved, the approach is not valid based on the loss of specificity that will occur at higher doses. Therefore, experiments were completed with the concentration limited to generate meaningful results.

Collectively these data suggest BMSC can be modulated by neurotrophins in a manner consistent with influence on hematopoietic cell proliferation and differentiation, reflected by a significant IL-6 increase in this study. As such, neurotrophins are positioned to regulate the availability of inflammatory cells derived from the marrow, through both direct[41]–[44] and indirect mechanisms. Taken together, our data suggest a central role for neurotrophins in the inflammatory process subsequent to infection and identify bone marrow stroma as a novel target for these factors. Further, this study broadens the context in which we should consider the consequences of dysregulated neurotrophin expression.

## Materials and Methods

### Ethics Statement

This study was conducted according to the principles expressed in the Declaration of Helsinki. The study was approved by the Institutional Review Board of West Virginia University. All patients provided written informed consent for the collection of samples and subsequent analysis.

### Cell culture and reagents

P163, P164, PED299, PED604, PED62304 and GPBM 1–32 primary human bone marrow stromal cells (BMSC) were derived from consenting donors (written consent) with the approval of the West Virginia University Institutional Review Board. Establishment of BMSC and their characterization have been previously described in detail[67]. Because the characteristics of BMSC can be influenced by preparative regimens, all lines were established identically and evaluated at comparable passage number in the experiments presented. BMSC were maintained in Minimum Essential Medium, Alpha (α-MEM) (Mediatech, Manassa, VA) supplemented with 10% fetal bovine serum (FBS) (Hyclone, Pittsburgh, PA), 2 mM L-glutamine (Mediatech) 100 mg/ml streptomycin, 100 IU/ml penicillin (Sigma, St. Louis, MO), and 5×10^−5^ M 2-β mercapthanol (Sigma), at 37°C in 6% CO_2_. Of note, the concentration of 2- β mercapthanol used is less than that published as having the potential to stimulate cells to neural differentiation[68]. The adherent, fibroblastic BMSC utilized in all of our studies constitutively express VCAM-1, Fibronectin, SDF-1 (CXCL-12), VEGF, Thrombospondin and a variety of cytokines that influence both lymphoid and myeloid cell survival and expansion.

### Flow cytometric analysis

Six different BMSC lines were grown to confluence (∼10^6^ cells), trypsinized, fixed in 10% formaldehyde for 30 minutes, and subsequently permeabilized in 70% EtOH for 30 minutes on ice. To reduce non-specific antibody binding, BMSC were blocked in 3% BSA in PBS for 15 minutes and subsequently incubated with 1 µg per sample of rabbit polyclonal TrkA or TrKB, goat polyclonal p75^NTR^ specific antibodies from Santa Cruz Biotechnology,Inc. (Santa Cruz, CA). For detection of TrkC, TrkC-PE (FAB373P) and isotype control goat IgG-PE were acquired from R&D systems (Minneapolis, MN). The additional isotype control antibody, rabbit IgG, was purchased from Southern Biotechnology (Birmingham, AL). Primary antibody binding was detected by incubation with 1 µg per sample fluorescein isothiocyanate (FITC)–conjugated goat anti-rabbit (Santa Cruz Biotechnology) for Trk A and TrkB and FITC–conjugated rabbit anti-goat IgG(H+L) (Southern Biotechnology) for p75NTR. Data were acquired by counting 10000 events and analyzed using FACSCalibur (BD Biosciences, San Jose, CA). One representative cell line from the six lines examined was chosen for completion of the remaining experiments in the manuscript unless indicated.

### Western blot analysis

Confluent BMSC were treated with 100 ng/ml mouse NGF 2.5 S (Roche Applied Science) or recombinant human BDNF (Invitrogen, Carlsbad, CA) for 5 minutes, 30 minutes, 1 hour and 6 hours. Following treatment, BMSC were lysed in complete cell lysis buffer (50 mM Tris-HCl [pH 7.4], 150 mM NaCl, 1% Triton X-100, 0.25% Na-deoxycholate, 1 mM EDTA, and 1 mM NaF, 1 mM DTT, 1 mM PMSF, 1 mM activated Na_3_VO_4_, 1 µg/mL aprotinin, 1 µg/mL leupeptin, and 1 µg/mL pepstatin) on ice for 15 minutes. Following centrifugation at 20,000×g for 15 minutes, supernatants were collected and protein concentration was determined using the bicinchoninic acid protein assay (BCA). Proteins were resolved on SDS-PAGE and transferred to nitrocellulose membranes (Schleicher & Schuell Bioscience, Keene, New Hampshire). Membranes were blocked in TBS/5% nonfat dry milk/0.1% Tween-20 at room temperature for 1 hour, and probed with the primary antibodies rabbit anti-phospho-Akt (Ser473) or rabbit anti-Akt, (Cell Signaling Technology, Inc, Danvers, MA). Additional antibodies included rabbit Erk 1/2 (Cell Signaling Technologies) and anti-phospho ERK1/2 purchased from Promega Corporation (Madison, WI). Mouse anti-GAPDH (Fitzgerald Industries International, Concord, MA) was used as a lane loading control. Washes were in TBS/0.1% Tween-20 following incubation with horseradish peroxidase-conjugated secondary antibodies. Luminol (Santa Cruz Biotechnology) generated signal was detected on x-ray film. Densitometric analysis was performed using the Fotodyne imaging system with Foto/Analyst version 5.00 software (FOTODYNE Inc., Hartland, WI) for image acquisition, and TotalLab version 2005 software for analysis.

### Cellular Fractionation and Western Blot Analysis

BMSC were left untreated or treated with NGF (100 ng/ml) for 30 min. Cells were trypsinized, pelleted and cellular fractionation was completed using the NE-PER cytoplasmic and nuclear extraction kit (Pierce Biotechnology, Rockford, IL). Protein concentration was determined using the bicinchoninic acid (BCA) protein assay (Pierce Biotechnology). Cytoplasmic and nuclear proteins were resolved on SDS-PAGE gels and transferred to nitrocellulose membranes. Membranes were blocked with 5% nonfat dry milk/1X TBS/0.1% Tween-20 and probed with a mouse monoclonal p65 specific antibody. After incubation with anti-mouse HRP- conjugated secondary antibody, the signal was visualized using Immobilon chemiluminescence reagents (Millipore, Billerica, MA).

### RNA Isolation

Total RNA was isolated from three BMSC lines treated with NGF or BDNF (100 ng/ml) using the Qiagen RNeasy Mini kit following the recommendations of the manufacturer (Qiagen, Valencia, CA). Pelleted BMSC were lysed by centrifugation through QIA shredder spin columns and RNA was treated with 1U DNAse for 15 minutes at 24°C. Samples were quantified at 260 nm (GENESYS-10UV, Spectronic, Unicom) and protein contamination determined by evaluation at 280 nm.

### Microarray analysis

Gene expression profiles of BMSC were assessed using the Human Neurotrophin and Receptor Gene Array HS-018 (SuperArray, Frederick, MD) as a representative, but not exhaustive, approach for screening of NGF or BDNF induced changes in gene expression. BMSC RNA from untreated control and NGF or BDNF treated cells was converted to biotinylated cDNA using the Ampolabeling-LPR kit (SuperArray). Membranes were hybridized with cDNA probes overnight at 60°C with continuous agitation at 5–10 r.p.m and then washed as recommended by the manufacturer. Signal was detected on X-ray film with images scanned and analyzed with GEArray Expression analysis suite software (SuperArray). Signal intensities were normalized to *GAPDH* and *beta-actin* on each membrane. Only those genes having a 3 fold or higher change in expression were examined (GEO accession number GSE18537).

### Real-time PCR

To determine relative IL-6 expression, real-time PCR was used to validate data from microarray experiments. The one-step QuantiTect SYBR Green RT-PCR Kit (Qiagen) was used as recommended by the manufacturer. All reactions were performed in triplicate using 80 ng of RNA per reaction, IL-6 gene primers (#PPH08958A; SuperArray) or the housekeeping gene *GUSB* (beta glucuronidase) (Real Time Primers, Elkins Park, PA). Amplifications were completed using a 7500 real-time PCR system (Applied Biosystems, Foster City, CA). Amplification conditions included 50°C for 30 minutes, 95°C for 15 min, 45 cycles of 94°C for 15 seconds, 55°C for 30 seconds, and 72°C for 45 seconds. The relative quantitative method (DDCT) was used to evaluate gene expression in experimental and control cells for each gene examined[69].

### ELISA

BMSC were cultured in a 96-well plate and allowed to grow to confluence. Primary BMSC exhibit contact inhibited grown upon reaching confluence and are grown to confluence to prevent any proliferation following treatments. When the BMSC were confluent, they were serum deprived (1% FBS) for 24 h before treatment with 100 ng/ml NGF or BDNF for 24 or 48 hours. Supernatants were collected and a human IL-6 ELISA completed (eBioscience, San Diego, CA) with 1/20 dilution of supernatants as recommended by the manufacturer. For experiments in which the effects of MEK and MAPK inhibition were studied, 20 µM of the MEK inhibitor U0126 or 10 µM of the p38 MAPK inhibitor SB 203580 (Promega) were added 2 hours before treatment with NGF or BDNF in select wells. Integrity of cell layers was confirmed prior to collection of supernatants. An MTT assay was completed to show that neurotrophin treatment did not induce proliferation or cell death (data not shown).

### Immunostaining and confocal microscopy

BMSC were grown to confluence on glass coverslips and treated with 100 ng/ml NGF, BDNF or rTNF-α (R&D Systems), for 5, 30 and 60 minutes. Following treatment, BMSC were rinsed in phosphate-buffered saline (PBS) and fixed in 4% formaldehyde at room temperature for 30 minutes, followed by rinsing and fixing in acetone for 10 minutes. Permeabilization of cells was completed with 0.5% Triton X-100 for 30 minutes. Nonspecific antibody binding was blocked by incubation of BMSC for 30 minutes in PBS/5% BSA. NF-κB localization was evaluated by incubation of BMSC with 2 µg/coverslip of mouse anti-human monoclonal NF-κB p65 (Santa Cruz Biotechnology) in PBS/5% BSA for 1 hour. Subsequently, Alexa Fluor 555 donkey anti-mouse IgG(H+L) (Invitrogen), was applied at a 1 µg/coverslip for one hour. Coverslips were then inverted on slides and mounted with Prolong Gold antifade reagent with 4, 6 diamidino-2-phenylindole (DAPI) (Invitrogen). Images were collected using a Zeiss LSM510 confocal on an AxioImager Z1 microscope (Carl Zeiss) with a 405-diode laser to excite DAPI and a 543 HeNe laser to excite the AlexaFluor 555-labeled secondary antibody. Cells were visualized using a 40×/1.30 oil objective. All confocal images were adjusted equally using Adobe Photoshop.

### Statistical analysis

Data presented were expressed as mean ± SEM for triplicate samples. Statistical analysis was performed by ANOVA (*p*≤0.01 determined as significant) and Student t-test (*p*≤0.01 determined as significant).

## References

[1] Levi-Montalcini R (1987). The nerve growth factor 35 years later.. Science.

[2] Barbacid M, Lamballe F, Pulido D, Klein R (1991). The trk family of tyrosine protein kinase receptors.. Biochim Biophys Acta.

[3] Boissiere F, Faucheux B, Ruberg M, Agid Y, Hirsch EC (1997). Decreased TrkA gene expression in cholinergic neurons of the striatum and basal forebrain of patients with Alzheimer's disease.. Exp Neurol.

[4] Siegel GJ, Chauhan NB (2000). Neurotrophic factors in Alzheimer's and Parkinson's disease brain.. Brain Res Brain Res Rev.

[5] Aloe L, Bracci-Laudiero L, Bonini S, Manni L (1997). The expanding role of nerve growth factor: from neurotrophic activity to immunologic diseases.. Allergy.

[6] Laudiero LB, Aloe L, Levi-Montalcini R, Buttinelli C, Schilter D (1992). Multiple sclerosis patients express increased levels of beta-nerve growth factor in cerebrospinal fluid.. Neurosci Lett.

[7] Raychaudhuri SP, Jiang WY, Farber EM (1998). Psoriatic keratinocytes express high levels of nerve growth factor.. Acta Derm Venereol.

[8] Aloe L, Skaper SD, Leon A, Levi-Montalcini R (1994). Nerve growth factor and autoimmune diseases.. Autoimmunity.

[9] Aloe L, Probert L, Kollias G, Bracci-Laudiero L, Spillantini MG (1993). The synovium of transgenic arthritic mice expressing human tumor necrosis factor contains a high level of nerve growth factor.. Growth Factors.

[10] DeKosky ST, Goss JR, Miller PD, Styren SD, Kochanek PM (1994). Upregulation of nerve growth factor following cortical trauma.. Exp Neurol.

[11] Washiyama K, Muragaki Y, Rorke LB, Lee VM, Feinstein SC (1996). Neurotrophin and neurotrophin receptor proteins in medulloblastomas and other primitive neuroectodermal tumors of the pediatric central nervous system.. Am J Pathol.

[12] Calza L, Giardino L, Aloe L (1997). NGF content and expression in the rat pituitary gland and regulation by thyroid hormone.. Brain Res Mol Brain Res.

[13] Bonini S, Lambiase A, Bonini S, Angelucci F, Magrini L (1996). Circulating nerve growth factor levels are increased in humans with allergic diseases and asthma.. Proc Natl Acad Sci U S A.

[14] Gordon MY, Goldman JM, Gordon-Smith EC (1983). Spatial and functional relationships between human hemopoietic and marrow stromal cells in vitro.. Int J Cell Cloning.

[15] Gordon MY, Kearney L, Hibbin JA (1983). Effects of human marrow stromal cells on proliferation by human granulocytic (GM-CFC), erythroid (BFU-E) and mixed (Mix-CFC) colony-forming cells.. Br J Haematol.

[16] Hirano T (1998). Interleukin 6 and its receptor: ten years later.. Int Rev Immunol.

[17] Kishimoto T (1989). The biology of interleukin-6.. Blood.

[18] Cromwell O, Hamid Q, Corrigan CJ, Barkans J, Meng Q (1992). Expression and generation of interleukin-8, IL-6 and granulocyte-macrophage colony-stimulating factor by bronchial epithelial cells and enhancement by IL-1 beta and tumour necrosis factor-alpha.. Immunology.

[19] Khair OA, Devalia JL, Abdelaziz MM, Sapsford RJ, Tarraf H (1994). Effect of Haemophilus influenzae endotoxin on the synthesis of IL-6, IL-8, TNF-alpha and expression of ICAM-1 in cultured human bronchial epithelial cells.. Eur Respir J.

[20] Hirano T, Matsuda T, Turner M, Miyasaka N, Buchan G (1988). Excessive production of interleukin 6/B cell stimulatory factor-2 in rheumatoid arthritis.. Eur J Immunol.

[21] Samoilova EB, Horton JL, Hilliard B, Liu TS, Chen Y (1998). IL-6-deficient mice are resistant to experimental autoimmune encephalomyelitis: roles of IL-6 in the activation and differentiation of autoreactive T cells.. J Immunol.

[22] Ohshima S, Saeki Y, Mima T, Sasai M, Nishioka K (1998). Interleukin 6 plays a key role in the development of antigen-induced arthritis.. Proc Natl Acad Sci U S A.

[23] Kishimoto T, Akira S, Taga T (1992). Interleukin-6 and its receptor: a paradigm for cytokines.. Science.

[24] Hirano T, Yasukawa K, Harada H, Taga T, Watanabe Y (1986). Complementary DNA for a novel human interleukin (BSF-2) that induces B lymphocytes to produce immunoglobulin.. Nature.

[25] Noma T, Mizuta T, Rosen A, Hirano T, Kishimoto T (1987). Enhancement of the interleukin 2 receptor expression on T cells by multiple B-lymphotropic lymphokines.. Immunol Lett.

[26] Suematsu S, Matsuda T, Aozasa K, Akira S, Nakano N (1989). IgG1 plasmacytosis in interleukin 6 transgenic mice.. Proc Natl Acad Sci U S A.

[27] Rodriguez MC, Bernad A, Aracil M (2004). Interleukin-6 deficiency affects bone marrow stromal precursors, resulting in defective hematopoietic support.. Blood.

[28] Faggioli L, Costanzo C, Donadelli M, Palmieri M (2004). Activation of the Interleukin-6 promoter by a dominant negative mutant of c-Jun.. Biochim Biophys Acta.

[29] Markel TA, Wang M, Crisostomo PR, Manukyan MC, Poynter JA (2008). Neonatal stem cells exhibit specific characteristics in function, proliferation, and cellular signaling that distinguish them from their adult counterparts.. Am J Physiol Regul Integr Comp Physiol.

[30] Furuno T, Nakanishi M (2006). Neurotrophic factors increase tumor necrosis factor-alpha-induced nuclear translocation of NF-kappaB in rat PC12 cells.. Neurosci Lett.

[31] May MJ, Ghosh S (1998). Signal transduction through NF-kappa B.. Immunol Today.

[32] Karin M, Cao Y, Greten FR, Li ZW (2002). NF-kappaB in cancer: from innocent bystander to major culprit.. Nat Rev Cancer.

[33] Handel ML, McMorrow LB, Gravallese EM (1995). Nuclear factor-kappa B in rheumatoid synovium. Localization of p50 and p65.. Arthritis Rheum.

[34] Schreiber S, Nikolaus S, Hampe J (1998). Activation of nuclear factor kappa B inflammatory bowel disease.. Gut.

[35] Hart LA, Krishnan VL, Adcock IM, Barnes PJ, Chung KF (1998). Activation and localization of transcription factor, nuclear factor-kappaB, in asthma.. Am J Respir Crit Care Med.

[36] Mattson MP, Camandola S (2001). NF-kappaB in neuronal plasticity and neurodegenerative disorders.. J Clin Invest.

[37] Schonhoff CM, Bulseco DA, Brancho DM, Parada LF, Ross AH (2001). The Ras-ERK pathway is required for the induction of neuronal nitric oxide synthase in differentiating PC12 cells.. J Neurochem.

[38] Zhuang ZY, Xu H, Clapham DE, Ji RR (2004). Phosphatidylinositol 3-kinase activates ERK in primary sensory neurons and mediates inflammatory heat hyperalgesia through TRPV1 sensitization.. J Neurosci.

[39] Freund-Michel V, Bertrand C, Frossard N (2006). TrkA signalling pathways in human airway smooth muscle cell proliferation.. Cell Signal.

[40] Brunet A, Datta SR, Greenberg ME (2001). Transcription-dependent and -independent control of neuronal survival by the PI3K-Akt signaling pathway.. Curr Opin Neurobiol.

[41] Matsuda H, Coughlin MD, Bienenstock J, Denburg JA (1988). Nerve growth factor promotes human hemopoietic colony growth and differentiation.. Proc Natl Acad Sci U S A.

[42] Kannan Y, Ushio H, Koyama H, Okada M, Oikawa M (1991). 2.5S nerve growth factor enhances survival, phagocytosis, and superoxide production of murine neutrophils.. Blood.

[43] Kannan Y, Matsuda H, Ushio H, Kawamoto K, Shimada Y (1993). Murine granulocyte-macrophage and mast cell colony formation promoted by nerve growth factor.. Int Arch Allergy Immunol.

[44] Hamada A, Watanabe N, Ohtomo H, Matsuda H (1996). Nerve growth factor enhances survival and cytotoxic activity of human eosinophils.. Br J Haematol.

[45] Boyle MD, Lawman MJ, Gee AP, Young M (1985). Nerve growth factor: a chemotactic factor for polymorphonuclear leukocytes in vivo.. J Immunol.

[46] Kobayashi H, Mizisin AP (2001). Nerve growth factor and neurotrophin-3 promote chemotaxis of mouse macrophages in vitro.. Neurosci Lett.

[47] Caneva L, Soligo D, Cattoretti G, De HE, Deliliers GL (1995). Immuno-electron microscopy characterization of human bone marrow stromal cells with anti-NGFR antibodies.. Blood Cells Mol Dis.

[48] Simone MD, De SS, Vigneti E, Papa G, Amadori S (1999). Nerve growth factor: a survey of activity on immune and hematopoietic cells.. Hematol Oncol.

[49] Schuhmann B, Dietrich A, Sel S, Hahn C, Klingenspor M (2005). A role for brain-derived neurotrophic factor in B cell development.. J Neuroimmunol.

[50] Li N, Yang H, Lu L, Duan C, Zhao C (2007). Spontaneous expression of neural phenotype and NGF, TrkA, TrkB genes in marrow stromal cells.. Biochem Biophys Res Commun.

[51] Rabizadeh S, Oh J, Zhong LT, Yang J, Bitler CM (1993). Induction of apoptosis by the low-affinity NGF receptor.. Science.

[52] Yoon SO, Casaccia-Bonnefil P, Carter B, Chao MV (1998). Competitive signaling between TrkA and p75 nerve growth factor receptors determines cell survival.. J Neurosci.

[53] Nakayama T, Mutsuga N, Tosato G (2007). Effect of fibroblast growth factor 2 on stromal cell-derived factor 1 production by bone marrow stromal cells and hematopoiesis.. J Natl Cancer Inst.

[54] Yu M, Zheng X, Witschi H, Pinkerton KE (2002). The role of interleukin-6 in pulmonary inflammation and injury induced by exposure to environmental air pollutants.. Toxicol Sci.

[55] Fattori E, Della RC, Costa P, Giorgio M, Dente B (1994). Development of progressive kidney damage and myeloma kidney in interleukin-6 transgenic mice.. Blood.

[56] Bracci-Laudiero L, Aloe L, Caroleo MC, Buanne P, Costa N (2005). Endogenous NGF regulates CGRP expression in human monocytes, and affects HLA-DR and CD86 expression and IL-10 production.. Blood.

[57] Piedimonte G (2003). Contribution of neuroimmune mechanisms to airway inflammation and remodeling during and after respiratory syncytial virus infection.. Pediatr Infect Dis J.

[58] Raychaudhuri SP, Raychaudhuri SK (2004). Role of NGF and neurogenic inflammation in the pathogenesis of psoriasis.. Prog Brain Res.

[59] Rameshwar P, Zhu G, Donnelly RJ, Qian J, Ge H (2001). The dynamics of bone marrow stromal cells in the proliferation of multipotent hematopoietic progenitors by substance P: an understanding of the effects of a neurotransmitter on the differentiating hematopoietic stem cell.. J Neuroimmunol.

[60] Nowicki M, Ostalska-Nowicka D, Kondraciuk B, Miskowiak B (2007). The significance of substance P in physiological and malignant haematopoiesis.. J Clin Pathol.

[61] Nowicki M, Ostalska-Nowicka D, Konwerska A, Miskowiak B (2006). The predicting role of substance P in the neoplastic transformation of the hypoplastic bone marrow.. J Clin Pathol.

[62] Elenkov IJ (2008). Neurohormonal-cytokine interactions: implications for inflammation, common human diseases and well-being.. Neurochem Int.

[63] Yamaguchi M, Kojima T, Kanekawa M, Aihara N, Nogimura A (2004). Neuropeptides stimulate production of interleukin-1 beta, interleukin-6, and tumor necrosis factor-alpha in human dental pulp cells.. Inflamm Res.

[64] Yamaguchi M, Ozawa Y, Mishima H, Aihara N, Kojima T (2008). Substance P increases production of proinflammatory cytokines and formation of osteoclasts in dental pulp fibroblasts in patients with severe orthodontic root resorption.. Am J Orthod Dentofacial Orthop.

[65] Sio SW, Puthia MK, Lu J, Moochhala S, Bhatia M (2008). The neuropeptide substance P is a critical mediator of burn-induced acute lung injury.. J Immunol.

[66] Scicchitano MS, McFarland DC, Tierney LA, Boyce RW, Frazier KS (2008). Role of p38 in regulation of hematopoiesis: effect of p38 inhibition on cytokine production and transcription factor activity in human bone marrow stromal cells.. Blood Cells Mol Dis.

[67] Gibson LF, Fortney J, Landreth KS, Piktel D, Ericson SG (1997). Disruption of bone marrow stromal cell function by etoposide 3.. Biol Blood Marrow Transplant.

[68] Woodbury D, Schwarz EJ, Prockop DJ, Black IB (2000). Adult rat and human bone marrow stromal cells differentiate into neurons.. J Neurosci Res.

[69] Livak KJ, Schmittgen TD (2001). Analysis of relative gene expression data using real-time quantitative PCR and the 2(-Delta Delta C(T)) Method.. Methods.

